# HIV-Specific Probabilistic Models of Protein Evolution

**DOI:** 10.1371/journal.pone.0000503

**Published:** 2007-06-06

**Authors:** David C. Nickle, Laura Heath, Mark A. Jensen, Peter B. Gilbert, James I. Mullins, Sergei L. Kosakovsky Pond

**Affiliations:** 1 Department of Microbiology, University of Washington School of Medicine, Seattle, Washington, United States of America; 2 Department of Biostatistics, University of Washington and Fred Hutchinson Cancer Research Center, Seattle, Washington, United States of America; 3 Department of Pathology, University of California, San Diego, La Jolla, California, United States of America; University of Oxford, United Kingdom

## Abstract

Comparative sequence analyses, including such fundamental bioinformatics techniques as similarity searching, sequence alignment and phylogenetic inference, have become a mainstay for researchers studying type 1 Human Immunodeficiency Virus (HIV-1) genome structure and evolution. Implicit in comparative analyses is an underlying model of evolution, and the chosen model can significantly affect the results. In general, evolutionary models describe the probabilities of replacing one amino acid character with another over a period of time. Most widely used evolutionary models for protein sequences have been derived from curated alignments of hundreds of proteins, usually based on mammalian genomes. It is unclear to what extent these empirical models are generalizable to a very different organism, such as HIV-1–the most extensively sequenced organism in existence. We developed a maximum likelihood model fitting procedure to a collection of HIV-1 alignments sampled from different viral genes, and inferred two empirical substitution models, suitable for describing between-and within-host evolution. Our procedure pools the information from multiple sequence alignments, and provided software implementation can be run efficiently in parallel on a computer cluster. We describe how the inferred substitution models can be used to generate scoring matrices suitable for alignment and similarity searches. Our models had a consistently superior fit relative to the best existing models and to parameter-rich data-driven models when benchmarked on independent HIV-1 alignments, demonstrating evolutionary biases in amino-acid substitution that are unique to HIV, and that are not captured by the existing models. The scoring matrices derived from the models showed a marked difference from common amino-acid scoring matrices. The use of an appropriate evolutionary model recovered a known viral transmission history, whereas a poorly chosen model introduced phylogenetic error. We argue that our model derivation procedure is immediately applicable to other organisms with extensive sequence data available, such as Hepatitis C and Influenza A viruses.

## Introduction

Nearly every computational and statistical method used for comparative gene sequence analysis employ a stochastic model for estimating rates of evolutionary change, either explicitly or implicitly. *A priori* knowledge about physical or chemical properties of nucleotide or amino acid residues can be used to define mechanistic models of substitutions. For example, the popular HKY85 [1] nucleotide substitution model estimates two separate substitution rates: one for *transitions*-substitutions between chemically similar purines (adenine and cytosine) or pyrimidines (guanine and thymine)-and one for transversions (all other substitutions). Universal evolutionary constraints form another basis for mechanistic model derivation. Codon models of Muse-Gaut [2] and Goldman-Yang [3] distinguished amino-acid altering (non-synonymous) and silent (synonymous) substitutions and have formed the basis of a popular and successful suite of methods for the analysis of selective pressures on coding sequences.

Existing literature on probabilistic models for protein sequences is extensive and spans several decades. One of the first of such models was the PAM (point accepted mutation) matrix [4]. A PAM matrix is derived from the inferred substitutions along a phylogenetic tree relating homologous sequences, by estimating the probability that any given amino acid residue in a protein will be replaced by any other residue after a pre-specified evolutionary interval. Other models based on observed sequence variability in large alignments of homologous protein sequences, such as the BLOSUM family [5], have proven popular and successful. Karlin and Ghandour [6] and George, Barker, and Hunt [7] proposed methods of weighting differences based on chemical, functional, charge and structural properties of amino acids and computing replacement probabilities based on the similarity of the involved residues. Doolittle's group proposed substitution matrices based on amino acid structural similarities combined with the ease of genetic interchange [8], while Stanfel added information pertaining to biochemical properties to inform the probability of amino acid interchangeability [9]. More recently, a generalized index of exchangeability based on a meta-analysis of empirical data has been suggested as a means of estimating the tolerability of particular amino acid exchanges [10]. A similar method based on pairwise amino-acid differences between homologous genes led to the derivation of the ambitiously named Universal Evolutionary Index [11]. A more statistically robust method for model inference incorporates phylogenetic likelihood [12], and infers substitution rates from a seed alignment, e.g., from mitochondrial sequences [13] or a sample from several protein families [14].

‘Generalist’ models that describe substitution patterns amalgamated from multiple genes and organisms may describe a particular organism or gene poorly. To date, there have been only a few ‘specialist’ models targeted to a particular gene [15], or genomic region [13]. In this manuscript we lay out a maximum likelihood framework and an accessible software implementation for estimating an organism/gene specific evolutionary model and alignment scoring matrix, describe common techniques for validating the model and infer a model from a large collection of HIV-1 sequences.

Reliable estimation of substitution rates from short sequences (e.g. 10Kb viral genomes) requires a substantial degree of sequence diversity, which may require millions of years to accumulate in vertebrates or plants. However, rapidly evolving retroviruses, with mutation rates of up to ∼10^6^ greater than that of vertebrates [16], [17], accumulate similar levels of divergence in a matter of years and are abundantly represented in public databases. Dimmic et al. [15] used a maximum likelihood procedure to estimate an amino acid substitution rate matrix for specific application to reverse transcriptase, a key retroviral polymerase protein that transcribes viral RNA into DNA suitable for integration into the host genome. However, we found that this model fitted HIV-1 data poorly, probably because HIV adopts organism-specific substitution biases, different from other retroviruses.

To improve the predictive accuracy of substitution matrices for HIV protein evolution, we estimated two stochastic models from multiple representative HIV-1 sequence alignments using maximum likelihood. The first model was derived from HIV data sampled from within individual patients (within individual, HIV within, HIV-W_m_). The second model was estimated from alignments where every sequence represented a population consensus from a patient (between individual, HIV between, HIV-B_m_). At first glance, one might question the need for two separate models, since within-patient evolution could simply be a shorter timescale version of the between-patient evolution. One argument against this intuitive deduction is that most of the substitutions generated in a given individual are selected against during or following transmission and therefore do not persist at the level of host populations [18]–[20], resulting in potentially discordant substitution patterns. For example, substitutions which enable the virus to escape the cellular immune response in a given host can be rapidly generated and fixed [21]. However, many of these substitutions carry a fitness cost in terms of lower replicative capacity and are not likely to persist upon transmission to an individual whose immune system does not target the same genetic region of the virus, obviating the need for a fitness-lowering substitution there [22]. Indeed, if there were no added benefit in considering two models, one would see similar fits to both within and between-host viral samples with both models. Our findings strongly argue against this scenario (see ‘Results’), showing that substitution patterns shaped by within-and between-host selective regimes are detectably different.

## Methods

### Preparation of reference sequence alignments

The HIV-W_m_ model was generated using aligned sequences derived from 48 patients (encompassing portions of the *env* gene from 32 patients, from the *pro* gene from 8, and from the *pol* gene from 7 patients), which contained a total of 6,328 pairwise amino acid differences. For the HIV-B_m_ model we used 8 data sets, described in Table 1, with a total of 7,189 amino acid differences. This number is far greater than the approximately 1,700 differences that were incorporated into the commonly used Dayhoff PAM matrix and nearly twice the number in the reference alignment used for the estimation of rtREV [15]

**Table 1:** Data sets used for the estimation of the between-host evolutionary process in HIV-1

Gene	Sites	Sequences (One per patient)	Length	
			Mean branch	Tree
gag	500	39	0.02	1.69
env gp120	463	107	0.047	9.87
env gp41	364	134	0.053	14.17
nef	202	117	0.034	7.88
pol	1003	43	0.018	1.52
rev exon 1	25	81	0.041	6.50
rev exon 2	90	171	0.032	11.01
tat	71	76	0.029	4.37
vpr	96	133	0.034	9.11
vpu	78	124	0.047	11.52

### Estimation of the substitution model

We adopted a maximum likelihood phylogenetic approach based on the procedure of Whelan and Goldman [14] to estimate the 190 evolutionary rates that define the general time reversible (REV/GTR) model of amino-acid substitution jointly from a set of sequence alignments. The substitution process is described by the rate matrix **Q**, whose entry *q_ij_* = *r_ij_π_j_* (for *i*,*j* = 1…20) defines the instantaneous rate of replacing residue *i* with residue *j*, i.e. the probability of substituting *i* with *j* over an infinitesimally small time interval Δ*t* is approximately *q_ij_*Δ*t*. Residues are numbered based on the alphabetical ordering of the standard IUPAC nomenclature. *π_j_*(*j* = 1…20) denotes the stationary frequency of residue *j*, estimated by the proportion of *j*, and *r_ij_* is the rate parameter. Making the standard assumption of time reversibility, we set *r_ij_* = *r_ji_*. In order to ensure that **Q** defines a proper Markov rate matrix, the standard constraint is applied to the diagonal elements: 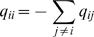.

The (*i,j*) entry of the matrix exponential *T*(*t*) = exp(**Q** *t*) defines the probability of replacing residue *i* with residue *j* in time *t*≥0. Because the likelihood function depends only on the products *q_ij_t*, one of the 190 rate parameters *r_ij_* is not identifiable. Following convention, we achieve identifiably by scaling the rate matrix so that the expected number of substitutions per amino-acid site per unit time, defined as 
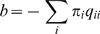
, is one.

The fitting algorithm proceeds as follows:

1. Given *N* codon alignments, we first reconstructed a neighbor joining tree [23] using the Tamura-Nei nucleotide distance metric [24] separately for each alignment. We decided against a more thorough method for topology reconstruction, partly for computational expediency and partly because rate estimation is thought to be fairly robust to small phylogenetic error. In addition, HIV-1 sequences often undergo recombination, and alignments with mosaic sequences cannot be properly described with a single phylogeny. To address this issue we carried out a screen for evidence of conflicting phylogenetic signal [25] and did not find strong evidence for phylogenetic discordance.
2. We estimated the equilibrium distribution of amino acid residues jointly from the N alignments and held it constant during the subsequent optimization procedure.
3. We used the HyPhy package [26] to perform a joint numerical optimization of branch lengths for each of the N alignments and the rates in the **Q** matrix. A flexible 5-bin *β*−Γ distribution [27] of rates across sites was included to account for the variation of substitution rates across sites. The three parameters of the distribution were shared by all N alignments. The numerical optimization algorithm in HyPhy can distribute multiple-dataset optimization across multiple nodes of a computer cluster, resulting in substantial optimization time reductions.
4. Because some of the 190 possible substitutions are rare, it is possible that the inference procedure estimates some of the *r_ij_* rates to be zero. However, fixing those estimates at zero for subsequent analyses is not biologically realistic, since it amounts to forcing some of the residue substitutions to always go through an intermediate state. For each rate *r_ij_*, whose maximum likelihood estimate was zero, we imputed the value of the rate using the following heuristic. If *S_i_* is the total number of protein sites in alignment *i* and *T_i_* is the total length of its fitted phylogenetic tree, we set 
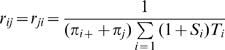
. This relation simply states that we would expect one *i*↔*j* substitution over all alignments given one more site per alignment.

### Performance on training data

We fitted newly estimated HIV models and three previously published models-JTT [28], WAG [14] and rtREV [15], estimating stationary frequencies from the data–to each of the training alignments. A comparison of model fits via standard techniques of likelihood ratio testing or AIC is complicated by the fact that because HIV models are estimated from the same sample that is being used to compare their fits to other models, it is difficult to correctly enumerate the effective number of rate parameters (and degrees of freedom) in HIV models when applied to a single training set. For a given training set, this number lies between 0 and 189 and depends on the contribution of the individual set to the joint rate matrix. We tabulated log-likelihood scores for each of the training data sets for reference purposes (Tables S1 and S2) and computed the number of additional degrees of freedom that an HIV model can support (at the 0.05 level) and still be preferred to each of the empirical models.

### Model validation

To ensure that our substitution models reflect true evolutionary patterns found in HIV-1 and are not fitting the noise in our training sets, we collected a number of independent HIV-1 sequence samples that were not used in model development across several genes, representing both within and between host sequences. 47 within patient samples (35 envelope glycoprotein (gp120) subtype B, 10 gp120 subtype C and 3 polymerase (*pol*) subtype B) and 11 between patient samples (representing *gag, pol, nef, env, vif, vpr, tat* and *rev* genes), covering both within- and between-subtype levels of diversity were collected. We note that if validation and training samples are not reciprocally monophyletic, shared evolution along internal tree branches may bias validation results. Within patient samples of HIV (assuming a single infection event/patient) drawn from different hosts are reciprocally monophyletic by definition, hence each alignment in the validation set forms an independent sample. All of our between-patient validation samples represent only non-B subtype non-recombinant viruses, and hence are reciprocally monophyletic with the training samples by definition of an HIV clade. We then fitted 19 models of protein evolution, including HIV-specific models, to each sample and ranked their performance using a small sample Akaike Information Criterion (c-AIC) score [29]–a robust measure of goodness of fit. We included six previously published empirical matrices: Dayhoff [4], JTT [28], WAG [14], rtREV [15], mtMAM [13] and mtREV 24 [30]. Each empirical model was examined both with the original model character frequency distribution (derived from the training set) and with frequencies gathered from the test data (conventionally referred to as the+F version of the model). We also fitted to each sample the 189 parameter reversible model (REV), and the restriction thereof (REV-1 model), which estimates separate rates between those amino-acids pairs that are one nucleotide substitution apart (75 pairs for the universal genetic code) and one shared rate for all multiple-nucleotide substitutions.

Additionally, we applied the Shimodaira-Hasegawa test to several example alignments to determine whether or not the improvement in goodness-of-fit was influenced by sampling variability. For example, it could be that most of the improvement in c-AIC scores is derived from a few ‘outlier’ sites (scenario sensitive to stochastic sampling) or that a majority of sites contribute to the improvement in c-AIC (scenario robust to stochastic sampling).

### Generation of a similarity matrix

Similarity or scoring matrices, such as the BLOSUM [5] family, assign to a pair of amino acids (a, b) a score that reflects how much more (or less) likely a homologous pair (a, b) is to occur when compared to a chance occurrence. The score can be estimated by 
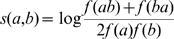
, where *f*(*a*) is the frequency of residue *a* in the reference set of aligned sequences, and *f*(*ab*) is the frequency of the pair computed from pairwise comparisons of homologous sequences in the reference set. The sum of all scores over alignment columns for a query pair of sequences serves as an approximation of the likelihood ratio statistic comparing the model of independence (null) with the model of homology (alternative).

Given a frequency distribution of amino-acid characters *π* and a transition matrix computed at time **T**(*t*_0_) = exp(**Q** *t*_0_), the score 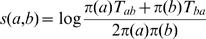 can be derived. The numerator lists the probability of evolving ‘b’ from ‘a’ or ‘a’ from ‘b’ in time *t*_0_, i.e., the probability of observing the (a,b) or (b,a) pair in two homologous sequences evolving under **Q**, while the denominator shows the probability of observing (a,b) or (b,a) in a pair of random sequences with residues drawn from the distribution *π*. The choice of *t*_0_ gives one control over how similar, on average, two sequences will be, much like the selection of more or less similar reference alignments gives rise to different BLOSUM and PAM matrices. Setting 
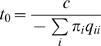
 will yield the expected sequence similarity of (1−*c*×100)%. Scores for aligning a character with a gap (‘-‘) can be adjusted case by case, or adopted from an existing model, such as BLOSUM62.

### Implementation

All the analyses reported here were implemented in the HyPhy software package. HyPhy scripts needed to fit a REV model to a collection of training alignments and generate similarity scoring matrices can be downloaded from http://www.hyphy.org/pubs/HIV-model/. HIV-1 models presented here are available as a part of the standard distribution of HyPhy (HIVWithin and HIVBetween models). Protein model comparisons can be carried out using the *AAModelComparison.bf* standard analysis in HyPhy. This implementation can also take advantage of a distributed cluster environment to accelerate rate estimation.

## Results

### Characterization of inferred rate matrices

**Figure 1:**
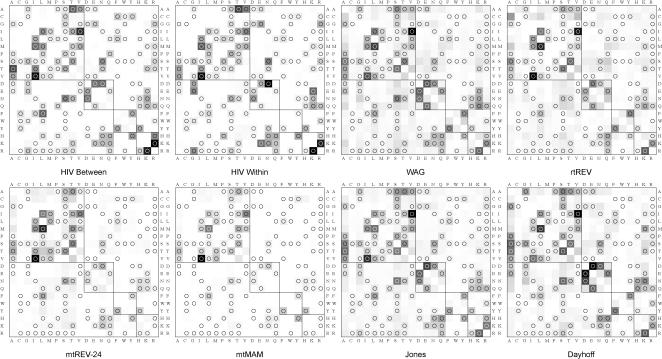
Rate matrices for different substitution models. All matrices are scaled to one expected substitution per unit time per site. Shading of the cells reflects the respective magnitude of the rate, with darker shades corresponding to increasingly higher rates. Substitutions which involve a single nucleotide are marked with a circle. The four diagonal blocks represent similarity classes (conservative substitutions) according to the Stanfel scale.

**Figure 2:**
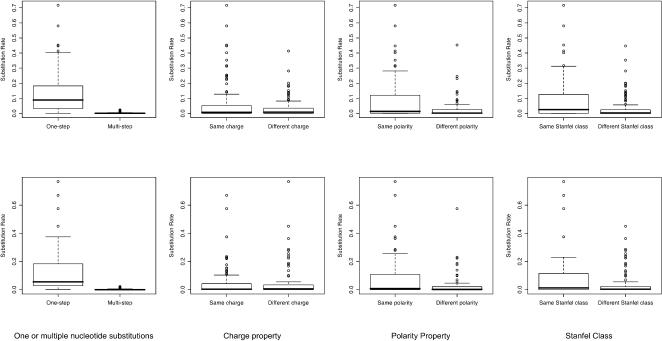
Inferred substitution rates. Rates are classified by whether or not a substitution involves single or multiple nucleotide changes, and by how they affect various properties of the residue being substituted. HIV-B_m_ model is plotted in the top row and HIV-W_m_ model-in the bottom row.

We first tabulated substitution rates inferred from the between-patient training set (**HIV-B_m_** model) and from the within-patient training set (**HIV-W_m_** model) and generated a graphical representation of six popular empirical matrices along with our two HIV specific matrices (Figure 1). Not surprisingly, most instantaneous substitution rates are low when compared to the highest rates in the matrix. Much like other empirical matrices, the HIV models assigned higher rates to those pairs that are separated by a single nucleotide substitution (see also Figure 2). However, there was little apparent correlation between higher substitution rates and preservation of a simple or complex physico-chemical property. Preservation or alteration of polarity, charge or similarity class, based on the classification scheme of Stanfel [9], had little effect on the median substitution rate (Figure 2), although among those few substitutions which had unusually high rates, more were conservative.

**Figure 3:**
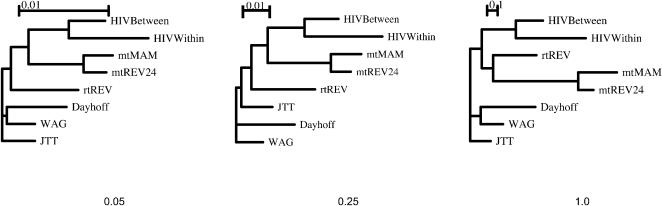
Model clustering using the Total Variation Metric at the evolutionary times equivalent to 5%, 25% and 100% sequence divergences.

Model-specific variability along with broad patterns of similarity are evident among all of the empirical models in Figure 1. To formally characterize the similarities in the substitution process across the eight matrices, we computed a neighbor-joining tree on the Markov processes defined by each matrix using the total variation metric (TVM) [31]. Briefly, given a specific evolutionary time scale, TVM computes the distance between the expected distributions of characters generated under the two evolutionary models. TVM distances take values in [0,1]. As expected, **HIV-B_m_** and **HIV-W_m_** models are most similar to each other over short (0.05 expected substitutions per site), medium (0.25) and long (1.0) evolutionary scales (Figure 3). In particular, HIV matrices define substitution patterns that are distinct from all other empirical models at several evolutionary time-scales. Somewhat surprisingly, the next closest set of matrices is that derived from mitochondrial sequences, except for the long evolutionary scale, when the rtREV model (derived from a viral reverse transcriptase protein alignment) becomes most similar to the HIV models. While counterintuitive, this finding seems less unexpected when one considers how the training sets were chosen for each of the models. Indeed, the Dayhoff, JTT, WAG and rtREV models were all trained on sequences that are much more heterogeneous, gearing them towards long-range evolutionary homologies.

### Model validation using independent data sets

To determine whether HIV Within and HIV Between models reflect evolutionary patterns found in circulating HIV-1 strains, rather than simply fitting the noise in the training sample, we tested model fits on samples of HIV-1 sequences not included in the training set. The results on samples collected from 47 different patients (the sequences from each patient formed a distinct test case) with a median of 32 sequences (range 10–57) and 357 sites (range 330–473), representing *env* and *pol* sequences from subtype B and subtype C viruses are shown in Tables 2 and 3. In 44/47 cases, the **HIV-W_m_** model with base frequencies estimated from the sample had the best small sample Akaike Information Criterion score (c-AIC) score of all 19 models compared. The c-AIC score of a model is defined as c-AIC = 2(−*L*+*ps*/(*s*−*p*−1)), where L is the log-likelihood score of the model, p is the number of estimated model parameters, and s is the number of independent samples. There are a number of possible ways to estimate the number of independent samples in the alignment [32] and we chose to use the number of alignment columns as an estimate of s. c-AIC performed well in selecting appropriate evolutionary models on biological and simulated alignments of paired RNA sequences [33]. In 2/47 cases, the **HIV-W_m_** model with the frequencies from the training set was the best and in 1/47 cases, the **HIV-B_m_** model was the best. In fact, the four HIV models predominantly occupied the four top ranks, suggesting that no other empirical model adequately represents the evolutionary rates shaping HIV-1 genomic variation. The REV-1 and JTT (+F) models were 5^th^ and 6^th^, respectively. Surprisingly, three models based on large heterogeneous database samples (JTT, WAG and mtMAM) outperformed the rtREV model, which was derived from a viral training dataset. Perhaps more importantly, our general HIV model estimated across many of the HIV genes consistently outperformed the rtREV model on sequence samples, including the HIV-1 reverse transcriptase protein.

**Table 2:** Relative performance of 19 protein models on a sample of 47 within-patient HIV-1 alignments^1^

Rank/Model	1	2	3	4	5	6	7	8	9	10	11	12	13	14	15	16	17	18	19
HIV-W_m_	44	2	0	1	0	0	0	0	0	0	0	0	0	0	0	0	0	0	0
HIV-W_m_+F	2	43	2	0	0	0	0	0	0	0	0	0	0	0	0	0	0	0	0
HIV-B_m_	1	0	18	16	6	3	2	1	0	0	0	0	0	0	0	0	0	0	0
HIV-B_m_+F	0	1	20	18	5	1	1	0	1	0	0	0	0	0	0	0	0	0	0
REV-1 step	0	1	3	0	2	2	3	3	5	4	1	7	1	1	3	6	1	4	0
JTT+F	0	0	4	7	22	12	2	0	0	0	0	0	0	0	0	0	0	0	0
JTT	0	0	0	5	9	21	10	1	1	0	0	0	0	0	0	0	0	0	0
WAG+F	0	0	0	0	1	7	22	14	2	1	0	0	0	0	0	0	0	0	0
mtMAM+F	0	0	0	0	1	0	1	2	1	1	2	6	1	4	14	13	1	0	0
rtREV	0	0	0	0	1	0	1	1	0	0	0	2	9	22	10	1	0	0	0
mtREV 24+F	0	0	0	0	0	1	0	6	6	4	8	7	11	4	0	0	0	0	0
WAG	0	0	0	0	0	0	5	13	18	5	3	1	2	0	0	0	0	0	0
Dayhoff+F	0	0	0	0	0	0	0	6	10	23	6	1	0	1	0	0	0	0	0
rtREV+F	0	0	0	0	0	0	0	0	2	4	14	14	12	1	0	0	0	0	0
Dayhoff	0	0	0	0	0	0	0	0	1	5	12	9	10	10	0	0	0	0	0
Equal Input	0	0	0	0	0	0	0	0	0	0	1	0	1	4	19	17	4	1	0
mtREV 24	0	0	0	0	0	0	0	0	0	0	0	0	0	0	1	7	37	2	0
mtMAM	0	0	0	0	0	0	0	0	0	0	0	0	0	0	0	2	3	39	3
REV	0	0	0	0	0	0	0	0	0	0	0	0	0	0	0	1	1	1	44

1 Based on small sample Akaike Information Criterion

**Table 3:** The number of times a model had evidence ratio of 100 or better against every competing model on the 47 within-patient validation data sets.^1^

	HIV-W_m_	HIV-W_m_+F	HIV-B_m_	HIV-B_m_+F	REV-1 step	JTT+F	JTT	WAG+F	MtMAM+F	rtREV	mtREV 24+F	WAG	Dayhoff+F	rtREV+F	Dayhoff	Equal Input	mtREV 24	mtMAM	REV
HIV-W_m_	0	45	44	46	47	46	47	47	47	46	47	47	47	47	47	47	47	47	47
HIV-W_m_+F	1	0	45	46	46	46	46	47	47	47	47	47	47	47	47	47	47	47	47
HIV-B_m_	0	1	0	15	43	30	39	43	46	46	46	46	46	47	47	47	47	47	47
HIV-B_m_+F	0	0	15	0	43	37	40	44	47	46	47	46	47	47	47	47	47	47	47
REV-1 step	0	1	4	4	0	6	6	11	31	32	22	14	17	24	28	35	41	43	47
JTT+F	0	0	8	5	40	0	28	47	46	46	47	47	47	47	47	47	47	47	47
JTT	0	0	3	3	38	4	0	35	44	46	45	47	47	46	47	47	47	47	47
WAG+F	0	0	3	1	34	0	5	0	43	44	43	39	42	46	47	47	47	47	47
MtMAM+F	0	0	0	0	16	0	0	2	0	14	2	6	4	7	12	31	47	47	46
rtREV	0	0	0	0	12	0	1	2	29	0	8	1	3	3	4	39	47	47	47
MtREV 24+F	0	0	0	0	18	0	1	1	41	37	0	7	7	22	25	47	47	47	47
WAG	0	0	0	1	29	0	0	2	40	45	35	0	30	39	43	46	47	47	47
Dayhoff+F	0	0	0	0	26	0	0	0	39	43	29	8	0	36	43	46	47	47	47
rtREV+F	0	0	0	0	19	0	0	0	35	41	20	2	1	0	20	46	47	47	47
Dayhoff	0	0	0	0	18	0	0	0	32	39	17	0	1	17	0	44	47	47	47
Equal Input	0	0	0	0	11	0	0	0	14	2	0	1	0	1	2	0	41	46	47
mtREV 24	0	0	0	0	4	0	0	0	0	0	0	0	0	0	0	4	0	43	45
mtMAM	0	0	0	0	4	0	0	0	0	0	0	0	0	0	0	0	1	0	44
REV	0	0	0	0	0	0	0	0	0	0	0	0	0	0	0	0	2	3	0

1 Models are arranged by decreasing rank performance (see Table 2)

The difference in c-AIC scores between two models can be interpreted as strength of evidence in favor of the model with the lower score. For instance, the evidence ratio for models A and B, R(A,B) is defined as *R*(*A,B*) = exp[(*AIC_C_*(*B*)−*AIC_C_*(*A*))/2], and can be interpreted as the relative probabilities of the two models generating the data in certain cases [34]. In all 47 cases the difference in c-AIC between **HIV-W_m_** and the best-fitting previously described empirical model was sufficiently large (median 125 points, range 13.3–259.5) to render the latter model not credible, with R(**HIV-W_m_**,best existing empirical model) >50 for every tested dataset. Furthermore, in 46/47 cases when **HIV-W_m_**(+F) outperformed **HIV-B_m_**(+F), R(**HIV-W_m_**, **HIV-B_m_**) was at most 0.011 (c-AIC difference range 9.0–215.3, median 94.4), confirming that a within-patient model for HIV is sufficiently different from the between-host model, supporting our argument for the need to derive these two distinct models. Table 3 provides a pairwise comparison of model performance by listing the number of times model A had evidence ratio in excess of 100 when compared to model B. For instance, **HIV-W_m_** had very strong evidence ratio support against all competing models in the vast majority of cases.

The results for 11 between-patient samples (where each sample contained a single sequence from each host), with a median of 37 sequences (range 22–119) and 442 sites (range 79–953) are shown in Tables 4 and 5. In 7/11 cases **HIV-B_m_** was the best fitting model (in all 11 cases it scored in the top 3), REV-1 was best in 4/11 cases. JTT consistently scored best among the existing empirical models, but always fit worse than **HIV-B_m_** (e.g., when it came in second, **HIV-B_m_** was first, see Table 5). In all 11 cases the difference in c-AIC between **HIV-B_m_** and the best-fitting previously described empirical model was sufficiently large (median 38.42 points, range 6.5–345.5) to suggest that previous empirical models fitted the data poorly, with R(**HIV-B_m_**,best existing empirical model) >25 for every tested dataset. In addition, **HIV-B_m_**, consistently outperformed **HIV-W_m_**, corroborating our original supposition for the need for two distinct models. The finding that the estimation of 76 rate parameters from the data was worthwhile in 4/11 cases is not surprising in retrospect. Some of the datasets we evaluated comprised multiple HIV subtypes with more variation and divergence than had been included in our subtype B-only training data. In fact, all 4 cases where REV-1 was found to be best, the sequences came from the Los Alamos National Laboratory HIV database subtype reference alignment spanning all common HIV-1 (clade M) subtypes. Furthermore, **HIV-B_m_** consistently outperformed all other empirical models.

**Table 4:** Relative performance of 19 protein models on a sample of 11 between-patient HIV-1 alignments^1^

Rank/Model	1	2	3	4	5	6	7	8	9	10	11	12	13	14	15	16	17	18	19
HIV-B_m_	7	2	1	1	0	0	0	0	0	0	0	0	0	0	0	0	0	0	0
REV-1 step	4	0	0	1	2	0	0	0	0	0	0	0	0	0	0	0	1	0	3
HIV-W_m_	0	5	2	1	1	1	0	1	0	0	0	0	0	0	0	0	0	0	0
JTT	0	2	3	3	0	0	1	0	1	1	0	0	0	0	0	0	0	0	0
HIV-B_m_+F	0	2	0	0	0	1	0	0	0	1	0	0	0	0	1	0	1	3	2
REV	0	0	3	1	1	0	3	1	1	0	0	1	0	0	0	0	0	0	0
JTT+F	0	0	1	1	4	1	1	1	1	1	0	0	0	0	0	0	0	0	0
WAG	0	0	1	0	2	0	0	0	1	0	0	3	2	1	1	0	0	0	0
Dayhoff	0	0	0	3	0	0	2	3	2	0	1	0	0	0	0	0	0	0	0
HIV-W_m_+F	0	0	0	0	1	3	1	2	0	1	2	1	0	0	0	0	0	0	0
mtMAM	0	0	0	0	0	4	0	1	0	4	1	1	0	0	0	0	0	0	0
rtREV	0	0	0	0	0	1	1	0	3	2	2	0	1	1	0	0	0	0	0
WAG+F	0	0	0	0	0	0	2	0	0	0	0	0	0	1	1	3	4	0	0
mtREV 24	0	0	0	0	0	0	0	2	0	0	0	0	0	0	0	1	1	6	1
mtREV 24+F	0	0	0	0	0	0	0	0	1	0	4	4	2	0	0	0	0	0	0
Dayhoff+F	0	0	0	0	0	0	0	0	1	0	1	1	4	2	2	0	0	0	0
rtREV+F	0	0	0	0	0	0	0	0	0	1	0	0	2	5	3	0	0	0	0
mtMAM+F	0	0	0	0	0	0	0	0	0	0	0	0	0	1	3	6	1	0	0
Equal Input	0	0	0	0	0	0	0	0	0	0	0	0	0	0	0	1	3	2	5

1 Based on small sample Akaike Information Criterion

**Table 5:** The number of times a model had evidence ratio of 100 or better against every competing model on the 11 between-patient validation data sets.^1^

	HIV-B_m_	REV-1 step	HIV-W_m_	JTT	HIV-B_m_+F	REV	JTT+F	WAG	Dayhoff	HIV-W_m_+F	mtMAM	rtREV	WAG+F	mtREV 24	mtREV 24+F	Dayhoff+F	rtREV+F	mtMAM+F	Equal Input
HIV-B_m_	0	7	9	10	9	11	11	11	11	11	11	11	11	11	11	11	11	11	11
REV-1 step	4	0	4	4	8	7	5	7	7	7	7	7	7	8	7	7	7	7	7
HIV-W_m_	0	6	0	5	9	9	8	11	10	10	11	10	11	11	11	11	11	11	11
JTT	0	7	5	0	9	8	8	8	8	11	8	11	9	9	11	11	11	11	11
HIV-B_m_+F	1	0	2	1	0	3	2	4	3	3	4	3	5	6	4	4	4	5	6
REV	0	4	2	3	8	0	5	10	6	7	9	10	11	11	11	10	11	11	11
JTT+F	0	6	2	0	9	6	0	8	8	10	8	10	9	9	11	11	11	11	11
WAG	0	4	0	3	7	1	3	0	0	3	3	4	11	11	4	6	10	11	11
Dayhoff	0	4	0	3	8	4	3	10	0	4	9	8	11	11	11	10	11	11	11
HIV-W_m_+F	0	4	1	0	8	3	1	7	5	0	6	8	9	9	11	11	10	11	11
mtMAM	0	4	0	2	7	2	3	8	2	4	0	6	11	11	9	11	11	11	11
rtREV	0	4	1	0	8	1	1	7	3	1	5	0	9	9	9	9	10	11	11
WAG+F	0	4	0	2	6	0	2	0	0	2	0	2	0	11	2	2	2	4	11
mtREV 24	0	3	0	2	5	0	2	0	0	2	0	2	0	0	2	2	2	2	9
mtREV 24+F	0	4	0	0	7	0	0	6	0	0	2	1	9	9	0	7	9	11	11
Dayhoff+F	0	4	0	0	7	1	0	3	1	0	0	2	9	9	1	0	8	10	11
rtREV+F	0	4	0	0	7	0	0	1	0	0	0	0	9	9	1	3	0	11	11
mtMAM+F	0	4	0	0	6	0	0	0	0	0	0	0	7	9	0	0	0	0	11
Equal Input	0	4	0	0	5	0	0	0	0	0	0	0	0	2	0	0	0	0	0

1 Models are arranged by decreasing rank performance (see Table 4)

### Sensitivity to stochastic sampling

We focus on a sample data set to investigate whether or not the improvement in model fit attained by HIV-specific models is driven by a few strong improvements at ‘outlier’ sites, or by consistent but smaller improvements at many sites in an alignment. A formal way to quantify the effect of stochastic sampling is to perform a Shimodaira-Hasegawa permutation test on columns of an alignment, compute the difference in log-likelihood scores of two competing models on each replicate (if they have the same number of parameters), and test the hypothesis that the difference is different from zero. We illustrate this on a sample of 119 *pol* sequences each sampled from a different host infected with subtype C HIV-1, with 951 sites each. The likelihood scores for three models on this alignment are as follows: **HIV-B_m_** log L = −21653.1, JTT log L = −21825.9, rtREV logL = −22383.4. Because the models have the same number of parameters, a better log likelihood leads to a better c-AIC score. When comparing **HIV-B_m_** to rtREV, we found that **HIV-B_m_** had higher likelihoods at 580/951 (61%) sites, with the median improvement of 0.99 (range of 0.002 to 37.9) log-likelihood points per site, whereas at the sites where rtREV performed better, the median difference was 0.7 (range 0–31.6). Based on 10,000 permutations, the SH test returned a p-value of <0.0001 in favor of **HIV-B_m_** having a better fit. In a comparison **HIV-B_m_** to JTT, the p-value in favor of **HIV-B_m_** was <0.018, **HIV-B_m_** fitted better on 557/951 (58.6%) sites, with the median improvement of 0.54 (0.0009–23.67) on those sites, and of 0.53 (0.00001–22.75) on those where JTT performed better. The largest improvement in favor of **HIV-B_m_** occurred at sites where there was significant polymorphism with 2 or 3 amino-acids estimated to have high substitution rates in HIV-1. For example, site 311 with base composition K_75_R_41_Q_2_G_1 _yielded improvements of over 20 log-likelihood points with HIV Between, when compared to JTT or rtREV. Note that **HIV-B_m_** assigned higher rates to K↔R than either JTT or rtREV, accommodating such variability (Figure 1).

### Effect of model misspecification on phylogenetic reconstruction

**Figure 4:**
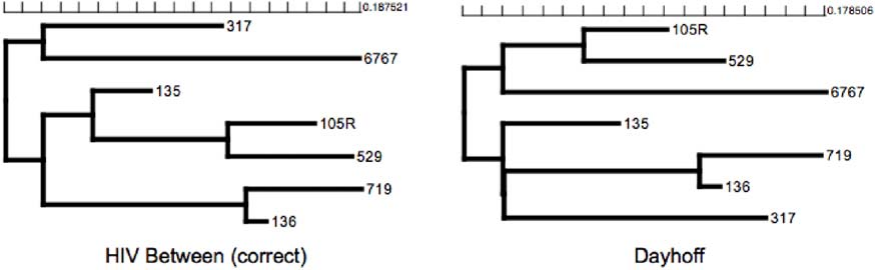
Maximum likelihood trees inferred with two different amino acid models from a sample of HIV-1 env V3 sequences with a known transmission history (Leitner et al. 1996). HIV-B_m_ found the tree that is congruent with the true history of the sequences. Scale bars are in expected amino acid substitutions/site.

We consider seven HIV-1 *env* V3 loop sequences sampled from an epidemiological cluster with known transmission history and hence a known phylogenetic tree, used as a study case by [35]. With seven sequences it is easy to perform an exhaustive search of all 945 unrooted trees and eliminate the influence of search heuristics. Thus any differences observed would be driven by model specification. The correct topology for the seven sequences is inferred by the **HIV-B_m_** model, but not by the Dayhoff model (Figure 4) for example. We note that better-fitting empirical models (e.g. JTT or WAG) recover the correct topology, and we use this example merely to illustrate that a poor or unjustified model choice may lead to erroneous results.

### Effect of model on evolutionary distance estimation

Model-based estimation of sequence divergence and diversity from sequence samples is a ubiquitous technique in HIV literature [36]. To examine the effect that an evolutionary model can have on the estimates of evolutionary distances between protein sequences we fitted four empirical models to 11 between-patient HIV alignments (Table S3). While median pairwise estimates (i.e. the total tree length) was not dramatically affected by the model choice (up to 5% relative error), for a particular pair of sequences, the estimates could vary by as much as ±30%.

### HIV similarity matrices

**Figure 5:**
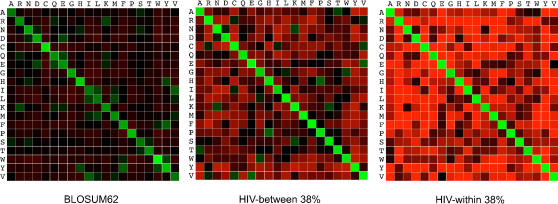
A comparison of BLOSUM and HIV-1 similarity scoring matrices with expected 62% sequence identity. Green = positive score; red = negative score; brightness = magnitude of score.

We compared our scoring matrices with the typically used BLOSUM62 scoring matrix with ours scaled to the same level of divergence (62% expected sequence identity). HIV matrices penalize (by assigning large magnitude negative scores) most kinds of substitutions more heavily than the BLOSUM62, perhaps representing the fact that long stretches of HIV-1 genomes are quite conserved (e.g. integrase). Figure 5 demonstrates the point. The BLOSUM62 matrix is relatively flat with many non-identities penalized relatively lightly and diagonal elements (identities) rewarded moderately. HIV matrices reward identities with higher scores than BLOSUM62 and tend to heavily penalize most non-identities.

## Discussion

We constructed two empirical HIV-1 subtype B amino acid substitution models to better describe patterns of HIV evolution, one based on among-patient sequences (HIV-B_m_), and the other based on within-patient sequences (HIV-W_m_). Our implementation is straightforward and relies on well-regarded methods of maximum likelihood phylogenetic inference. Indeed, a researcher can build an empirical amino acid model for any taxon or clade of choice assuming there is sufficient sequence data to estimate most of the rates reliably. In a direct comparison between the two HIV models, we find them to be similar, yet in our cross validation study we find that the HIV-W_m_ fits significantly better to within-patient data than the HIV-B_m_ models and vice versa. But most importantly, our HIV specific models fit dramatically better than any commonly used amino acid models, and nearly always better than parameter rich reversible models which estimate rates directly from the data set being tested. This finding suggests that empirical HIV models are generalizable to independent samples of HIV and can be recommended as the default matrices for comparative HIV analyses. Interestingly, the JTT model provided the best fit to our samples of HIV data among existing empirical models, which is in direct conflict with the observations made by Dimmic et al. [15].

A possible component of poor fit shown by existing empirical matrices on HIV sequences is the assumption that the sequences being compared have the same average amino acid composition as that used in producing the model. Because HIV sequences almost certainly do not have the same amino acid frequencies as those built into prior empirical matrices, the inclusion of HIV specific residue frequencies can be expected to improve the models. However, this is not the sole determinant of better model fits, because allowing for HIV-specific residue composition in existing empirical models (+F) failed to match the improvement in fit garnered by the use of HIV-specific substitution rates.

The lack of strong correlation between substitution rate and simple amino acid properties may be explained by the fact that because these models average rates over sites in different genes, which are subject to varying functional constraints, no single property can be expected to explain tremendous local variation well. This is true of other empirical models as well, as evidenced by the large number of shaded (high rate) pairs outside diagonal boxes, which reflect radical substitutions according to the Stanfel scale (Figure 1).

HIV specific models will improve the accuracy in measurements of evolutionary distance and phylogenetic inference on HIV sequences. For example, our models identify the correct phylogeny in a known transmission chain [35], whereas a poorly chosen model does not. The HIV-specific protein models of evolution could be used to tailor drug therapies against strains of viruses that maximize the protein distance that the virus would have to evolve to develop drug resistance. The use of more accurate evolutionary models can be used to improve the design of candidate vaccine strains, particularly those based on computationally derived sequences (e.g. Center-Of-Tree (COT) [37]). The models also have applications for comparing amino acid frequencies and patterns between sequence sets, and for generating HIV-like sequence data sets in computer simulation studies. In this manuscript we focused on organism-wide substitution patterns and our validation process convincingly showed that HIV models outperformed existing empirical models on a variety of individual gene alignments. Further possible refinements could include gene specific matrices for commonly sequenced viral genes such as *pol* or *env*.

The HIV-specific scoring matrices provided here should improve HIV sequence similarity searching (e.g. BLAST) and alignment (e.g. CLUSTALW). All sequence analyses that produce an alignment use a scoring matrix (e.g. BLOSUM) to weight the probability of an observed substitution. Sometimes these matrices are uninformative and treat all substitutions equally likely, but nearly all protein sequence based procedures use a matrix that ranks certain types of substitutions as more tolerable than others. We developed an efficient computational tool to facilitate the estimation of substitution matrices from training alignments and subsequent generation of scoring matrices at the desired level of evolutionary distance. We believe that with readily accessible modern computing power, it is now feasible to estimate and use organism specific empirical matrices for protein sequence analysis using powerful statistical techniques. Increased accuracy in comparative analyses, in our minds, is well worth the additional effort.

## Supporting Information

Table S1 Relative performance of HIV-B^m^ and three empirical models on between-host training data. Relative D.F. shows the number of additional degrees of freedom that HIV-B^m^ can have and still be preferred (by nested LRT at p = 0.05) to a given empirical model (see text).
(0.03 MB DOC)

Table S2 Relative performance of HIV-W^m^ and three empirical models on between-host training data. Relative D.F. shows the number of additional degrees of freedom that HIV-W^m^ can have and still be preferred (by nested LRT at p = 0.05) to a given empirical model (see text).
(0.10 MB DOC)

Table S3 The effect of evolutionary model on pairwise distance estimates using 11 between patient datasets. HIV-B^m^ is used as a reference model to compute tree-based pairwise distances, and relative differences for 3 existing empirical models are shown for each dataset.
(0.04 MB DOC)
