# Supplementary material for: HIV-Specific Probabilistic Models of Protein Evolution
Source: PLoS One. 2007 Jun 6;2(6):e503. doi: 10.1371/journal.pone.0000503 (PMC1876811; doi:10.1371/journal.pone.0000503)
Supplement: Table S1 — Relative performance of HIV-Bm and three empirical models on between-host training data. Relative D.F. shows the number of additional degrees of freedom that HIV-Bm can have and still be preferred (by nested LRT at p = 0.05) to a given empirical model (see text). (0.03 MB DOC) [file pone.0000503.s001.doc]

| Dataset | **HIV-B_m_** | **JTT+F** | | **WAG+F** | | **rtREV+F** | |
| --- | --- | --- | --- | --- | --- | --- | --- |
|  | LogL | LogL | Relative D.F. | LogL | Relative D.F. | LogL | Relative D.F. |
| **1** | -5799.49 | -5847.48 | 74.7915 | -5972.87 | >189 | -5971.35 | >189 |
| **2** | -22909.5 | -23344.2 | >189 | -23630.9 | >189 | -24072.6 | >189 |
| **3** | -17497.8 | -17987.3 | >189 | -18412.7 | >189 | -18648.6 | >189 |
| **4** | -9298.18 | -9425.6 | >189 | -9602.51 | >189 | -9708.2 | >189 |
| **5** | -10359.4 | -10488.6 | >189 | -10718.6 | >189 | -10748.8 | >189 |
| **6** | -720.208 | -739.481 | 25.7245 | -756.006 | 53.5194 | -762.302 | 64.4475 |
| **7** | -5643.1 | -5883.39 | >189 | -6106.65 | >189 | -6244.68 | >189 |
| **8** | -2047.96 | -2079.09 | 45.5143 | -2109.66 | 99.1565 | -2176.95 | >189 |
| **9** | -3281.79 | -3351.01 | 112.665 | -3444.61 | >189 | -3501.97 | >189 |
| **10** | -4173.39 | -4266.87 | 156.745 | -4356.63 | >189 | -4350.76 | >189 |
