# Supplementary material for: HIV-Specific Probabilistic Models of Protein Evolution
Source: PLoS One. 2007 Jun 6;2(6):e503. doi: 10.1371/journal.pone.0000503 (PMC1876811; doi:10.1371/journal.pone.0000503)
Supplement: Table S2 — Relative performance of HIV-Wm and three empirical models on between-host training data. Relative D.F. shows the number of additional degrees of freedom that HIV-Wm can have and still be preferred (by nested LRT at p = 0.05) to a given empirical model (see text). (0.10 MB DOC) [file pone.0000503.s002.doc]

| Dataset | **HIV-W_m_** | **JTT+F** | | **WAG+F** | | **rtREV+F** | |
| --- | --- | --- | --- | --- | --- | --- | --- |
|  | LogL | LogL | Relative D.F. | LogL | Relative D.F. | LogL | Relative D.F. |
| **1** | -3064.09 | -3146.67 | 136.861 | -3209.12 | >189 | -3253.79 | >189 |
| **2** | -3071.19 | -3149.67 | 129.409 | -3216.17 | >189 | -3260.28 | >189 |
| **3** | -2812.05 | -2881.88 | 113.766 | -2922.87 | 188.598 | -2952.93 | >189 |
| **4** | -2466.66 | -2525.42 | 93.909 | -2552.89 | 143.504 | -2591.12 | >189 |
| **5** | -1441.63 | -1497.88 | 89.4169 | -1522.9 | 134.463 | -1551.66 | 187.125 |
| **6** | -3132.57 | -3235.68 | 174.413 | -3280.18 | >189 | -3329.49 | >189 |
| **7** | -3587.15 | -3701.71 | >189 | -3775.77 | >189 | -3852.56 | >189 |
| **8** | -2912.06 | -3009.35 | 163.724 | -3055.55 | >189 | -3101.1 | >189 |
| **9** | -1799.69 | -1873.81 | 121.51 | ->1899.64 | 168.599 | -1933.06 | >189 |
| **10** | -1754.57 | -1789.67 | 52.3078 | -1806.77 | 82.2222 | -1844.83 | 150.849 |
| **11** | -2577.53 | -2623.64 | 71.4845 | -2653.91 | 125.605 | -2709.43 | >189 |
| **12** | -523.586 | -529.402 | 5.36563 | -533.332 | 10.8662 | -538.174 | 18.2415 |
| **13** | -1003.72 | -1014.03 | 11.6962 | -1027.35 | 32.8822 | -1029.25 | 36.0452 |
| **14** | -2134.16 | -2186.14 | 81.8365 | -2203.04 | 112.047 | -2257 | >189 |
| **15** | -394.034 | -395.028 | 0.380902 | -400.738 | 6.55012 | -400.787 | 6.61599 |
| **16** | -1027.17 | -1022.7 | 0 | -1032.47 | 4.70158 | -1028.08 | 0.341162 |
| **17** | -4072.63 | -4160.37 | 146.243 | -4200.89 | >189 | -4301.82 | >189 |
| **18** | -512.578 | -515.673 | 2.10298 | -521.462 | 9.61008 | -517.867 | 4.68424 |
| **19** | -1689.08 | -1706.32 | 22.4364 | -1745.9 | 90.4295 | -1744.97 | 88.7749 |
| **20** | -1057.93 | -1086.45 | 41.0895 | -1102.05 | 67.9904 | -1118.71 | 97.5129 |
| **21** | -336.156 | -337.859 | 0.830937 | -340.047 | 2.98165 | -339.82 | 2.72323 |
| **22** | -1297.37 | -1299.8 | 1.44883 | -1322.97 | 36.1822 | -1316.23 | 25.056 |
| **23** | -1160.71 | -1176.48 | 20.0922 | -1188.64 | 40.083 | -1207.33 | 72.3736 |
| **24** | -634.117 | -643.699 | 10.6255 | -650.503 | 21.0817 | -657.546 | 32.5498 |
| **25** | -1556.72 | -1576.16 | 25.9818 | -1606.26 | 77.5115 | -1615.78 | 94.4276 |
| **26** | -1584.63 | -1614.35 | 43.1195 | -1622.79 | 57.5956 | -1648.54 | 103.111 |
| **27** | -438.654 | -446.014 | 7.44955 | -451.104 | 14.9262 | -451.394 | 15.3709 |
| **28** | -1072.28 | -1081.63 | 10.2776 | -1093.63 | 29.1102 | -1092.39 | 27.0759 |
| **29** | -4655.73 | -4841.34 | >189 | -4947.32 | >189 | -5064.19 | >189 |
| **30** | -1160.14 | -1184.1 | 33.4341 | -1198.05 | 57.1839 | -1211.79 | 81.2591 |
| **31** | -384.014 | -386.949 | 1.93794 | -391.503 | 7.62986 | -391.66 | 7.85013 |
| **32** | -1001.07 | -994.534 | 0 | -1001.67 | 0.205376 | -1011.86 | 12.4085 |
| **33** | -2168.09 | -2229.6 | 98.812 | -2258.79 | 151.672 | -2300.62 | >189 |
| **34** | -7167.63 | -7437.21 | >189 | -7612.45 | >189 | -7790.74 | >189 |
| **35** | -2898.28 | -3012.37 | >189 | -3063.32 | >189 | -3113.13 | >189 |
| **36** | -2988.33 | -3107.96 | >189 | -3170.56 | >189 | -3250.56 | >189 |
| **37** | -4885.68 | -5055.85 | >189 | -5110.91 | >189 | -5261.97 | >189 |
| **38** | -4078.38 | -4280.36 | >189 | -4413.25 | >189 | -4499.58 | >189 |
| **39** | -737.727 | -745.172 | 7.56805 | -751.386 | 16.7904 | -756.869 | 25.511 |
| **40** | -1069.43 | -1110.13 | 62.0199 | -1126.86 | 91.5265 | -1134.5 | 105.199 |
| **41** | -1337.93 | -1383.79 | 71.0461 | -1398.18 | 96.5543 | -1424.54 | 144.194 |
| **42** | -805.897 | -822.927 | 22.1087 | -833.064 | 38.7995 | -838.591 | 48.1846 |
| **43** | -1376.88 | -1430.97 | 85.5782 | -1457.45 | 133.194 | -1481.37 | 176.939 |
| **44** | -1117.2 | -1126.98 | 10.9128 | -1134.06 | 21.8446 | -1137.71 | 27.7488 |
| **45** | -727.498 | -736.597 | 9.9216 | -745.904 | 24.3204 | -747.485 | 26.887 |
| **46** | -3391.82 | -3506.82 | >189 | -3576.71 | >189 | -3655.04 | >189 |
| **47** | ->1899.74 | -1971.49 | 117.229 | -1994.06 | 158.289 | -2015.98 | >189 |
| **48** | -319.973 | -324.062 | 3.2112 | -326.042 | 5.69861 | -325.979 | 5.61585 |
| **49** | -851.893 | -853.889 | 1.06127 | -858.646 | 6.61641 | -858.315 | 6.16849 |
