# Supplementary material for: HIV-Specific Probabilistic Models of Protein Evolution
Source: PLoS One. 2007 Jun 6;2(6):e503. doi: 10.1371/journal.pone.0000503 (PMC1876811; doi:10.1371/journal.pone.0000503)
Supplement: Table S3 — The effect of evolutionary model on pairwise distance estimates using 11 between patient datasets. HIV-Bm is used as a reference model to compute tree-based pairwise distances, and relative differences for 3 existing empirical models are shown for each dataset. (0.04 MB DOC) [file pone.0000503.s003.doc]

| Alignment | HIV-B_m_ | | | Relative JTT+F | | | | | Relative WAG+F | | | | | Relative rtREV+F | | | | |
| --- | --- | --- | --- | --- | --- | --- | --- | --- | --- | --- | --- | --- | --- | --- | --- | --- | --- | --- |
|  | Median | 2.5% | 97.5% | Median | | 2.5% | 97.5% | | Median | | 2.5% | 97.5% | | Median | | 2.5% | 97.5% | |
| Subtype A gp120 | 22.47% | 17.27% | 28.46% | 2.31% | -4.97% | | | 9.96% | 0.98% | -6.97% | | | 9.04% | 3.22% | -5.00% | | | 11.51% |
| Subtype A RT | 10.31% | 2.03% | 16.95% | 0.75% | -8.36% | | | 9.60% | 0.69% | -8.41% | | | 8.40% | 0.69% | -8.60% | | | 9.25% |
| Subtype C pol | 7.15% | 4.89% | 9.72% | 1.98% | -2.93% | | | 7.67% | 2.05% | -3.19% | | | 8.16% | 3.15% | -3.91% | | | 10.49% |
| Subtype D RT | 9.77% | 4.44% | 20.90% | 1.50% | -4.17% | | | 10.42% | 1.56% | -5.20% | | | 11.56% | 1.56% | -5.91% | | | 9.92% |
| Subtype D gag | 12.73% | 6.39% | 20.51% | -1.20% | -6.00% | | | 1.74% | -1.49% | -6.33% | | | 2.09% | -0.24% | -3.77% | | | 2.86% |
| Group M  gag | 24.68% | 10.13% | 31.04% | -1.82% | -6.00% | | | 1.78% | -1.94% | -6.54% | | | 2.17% | -0.18% | -4.38% | | | 3.89% |
| Group M  pol | 13.21% | 5.47% | 16.07% | 0.48% | -2.47% | | | 3.52% | 0.06% | -2.98% | | | 3.57% | 0.70% | -2.81% | | | 4.34% |
| Group M  vif | 30.26% | 11.04% | 39.16% | 2.43% | -7.31% | | | 10.16% | 2.51% | -7.67% | | | 10.33% | 3.09% | -6.96% | | | 11.24% |
| Group M  vpr | 16.22% | 6.50% | 28.11% | -3.03% | -18.56% | | | 19.36% | -3.08% | -17.76% | | | 20.81% | -1.86% | -17.04% | | | 27.01% |
| Group M  tat | 49.62% | 17.32% | 70.44% | -4.47% | -13.51% | | | 2.91% | -5.23% | -18.33% | | | 8.09% | -2.41% | -13.81% | | | 6.00% |
| Group M  rev | 40.46% | 17.47% | 61.17% | -5.45% | -12.74% | | | 2.94% | -5.92% | -14.14% | | | 2.85% | -4.09% | -13.08% | | | 5.47% |
